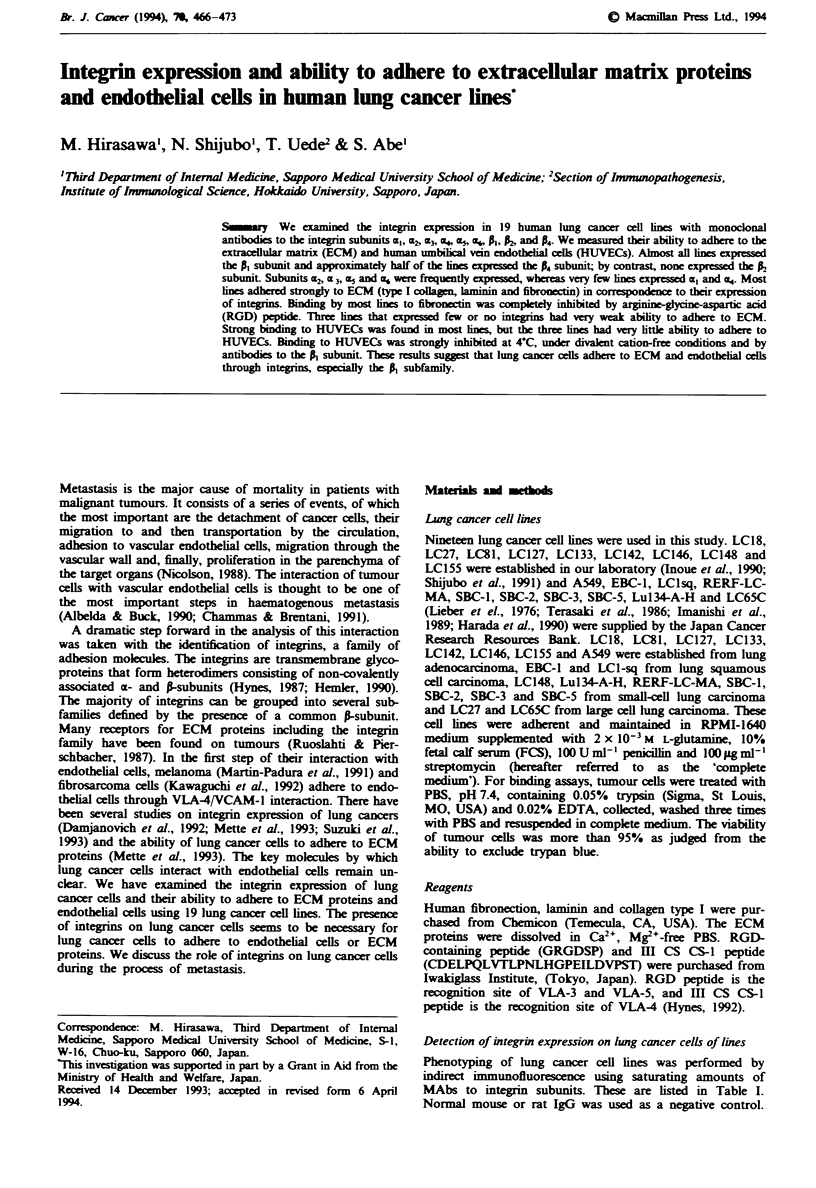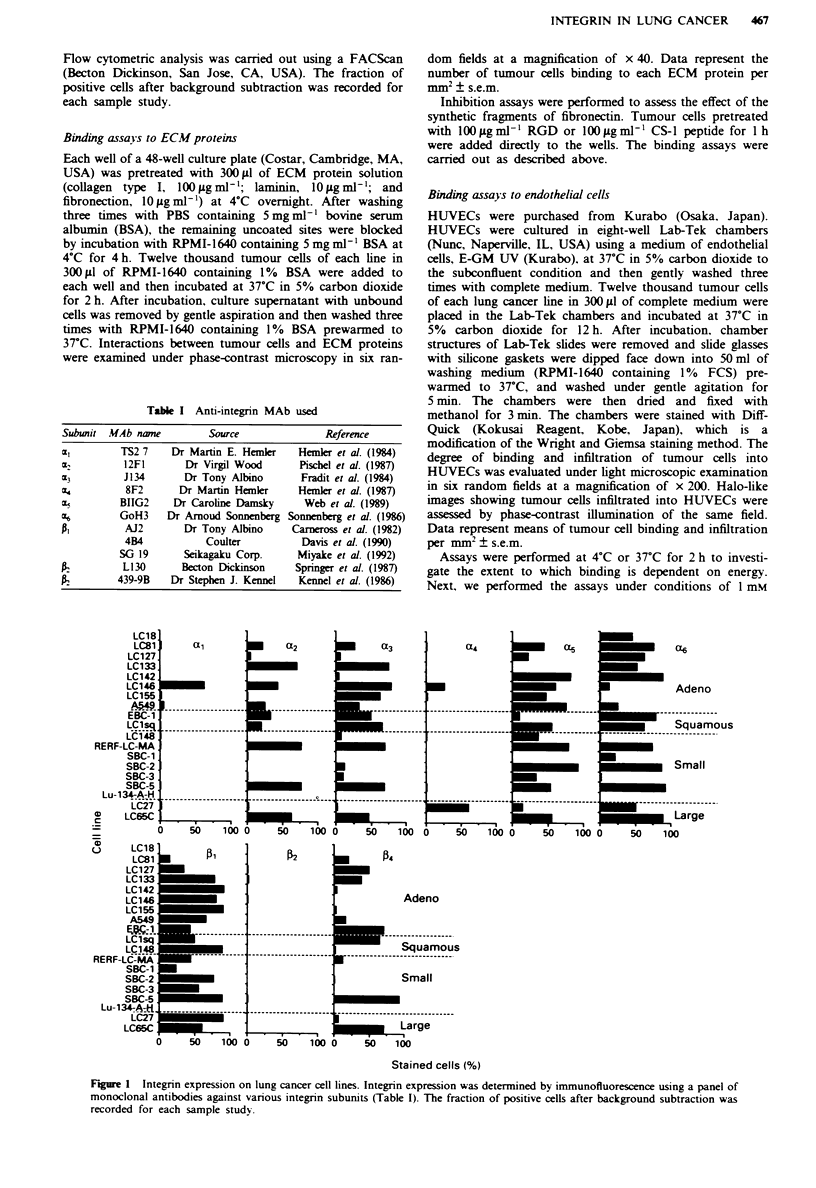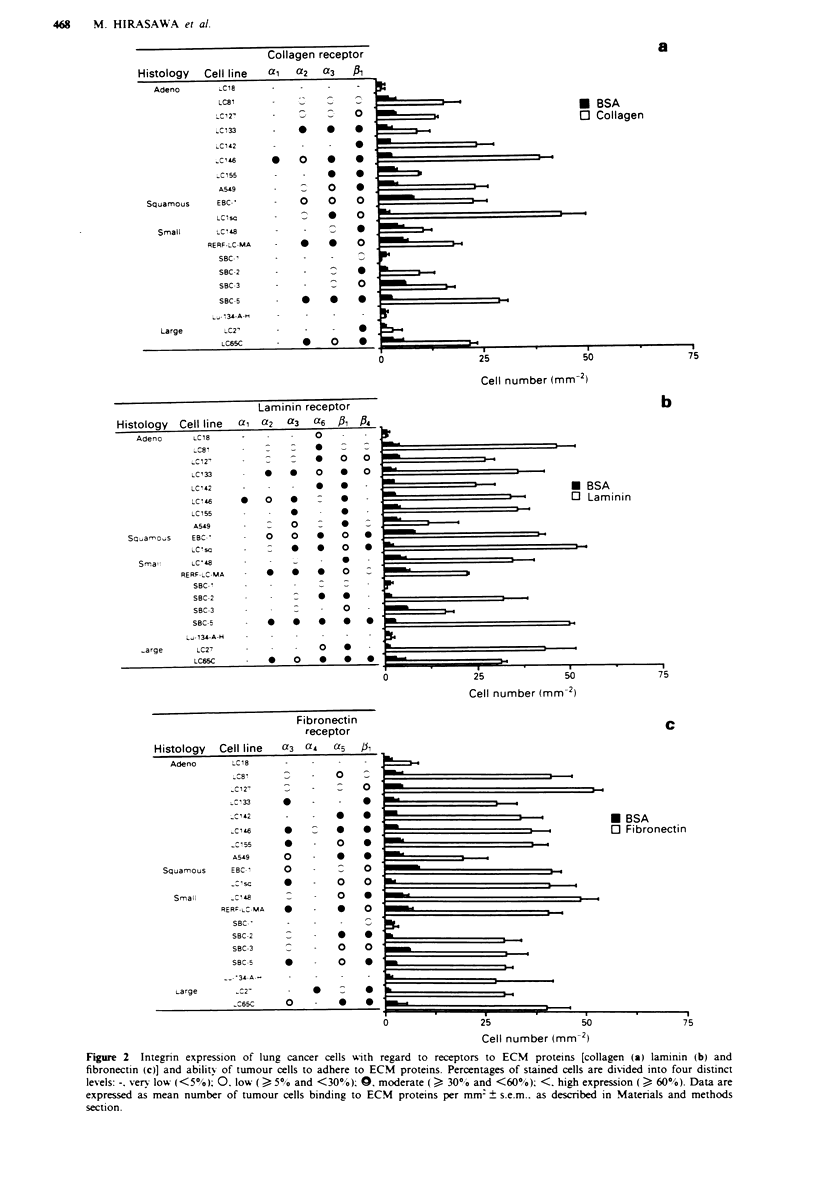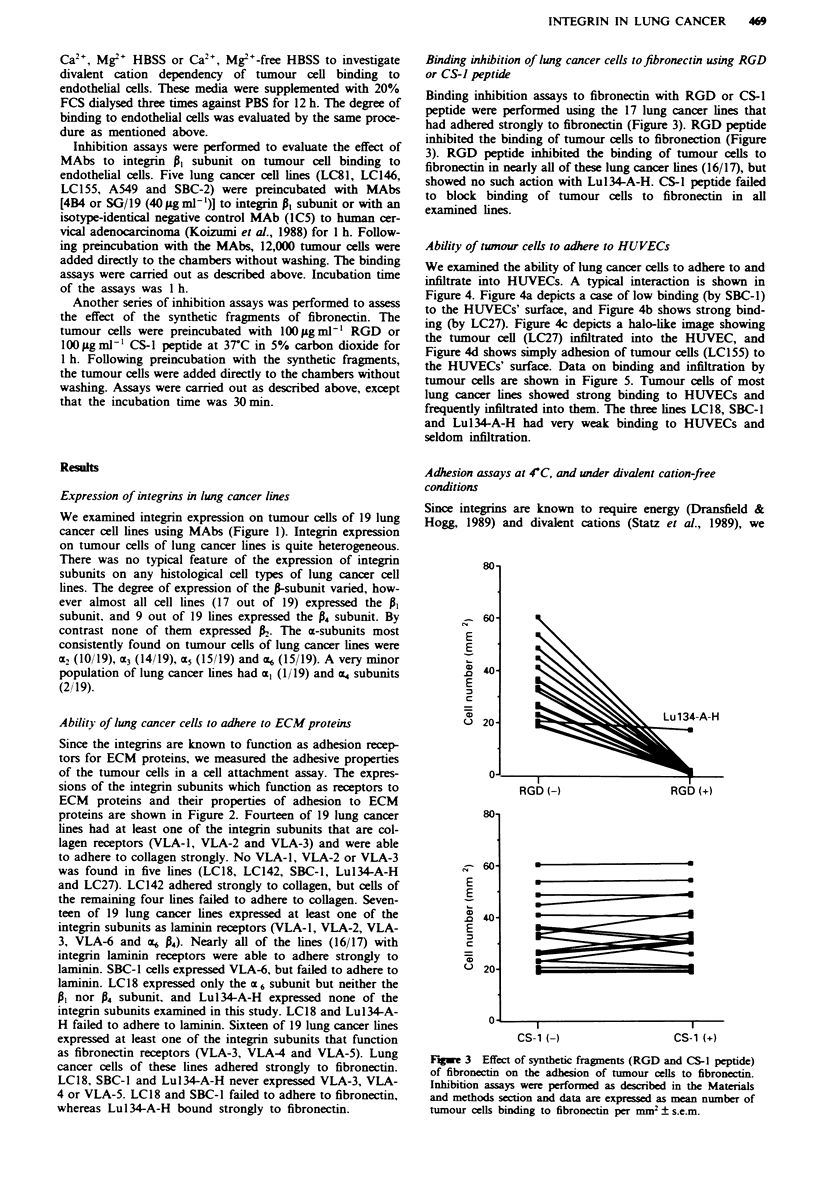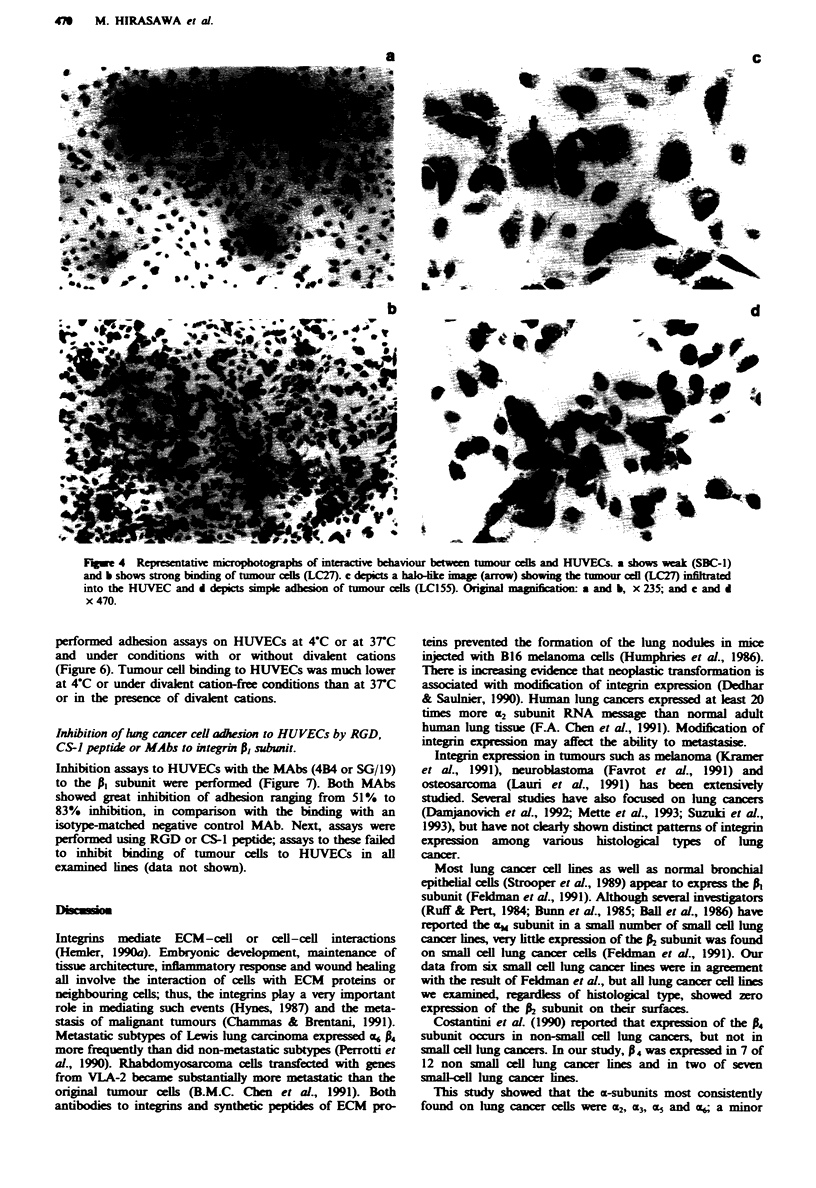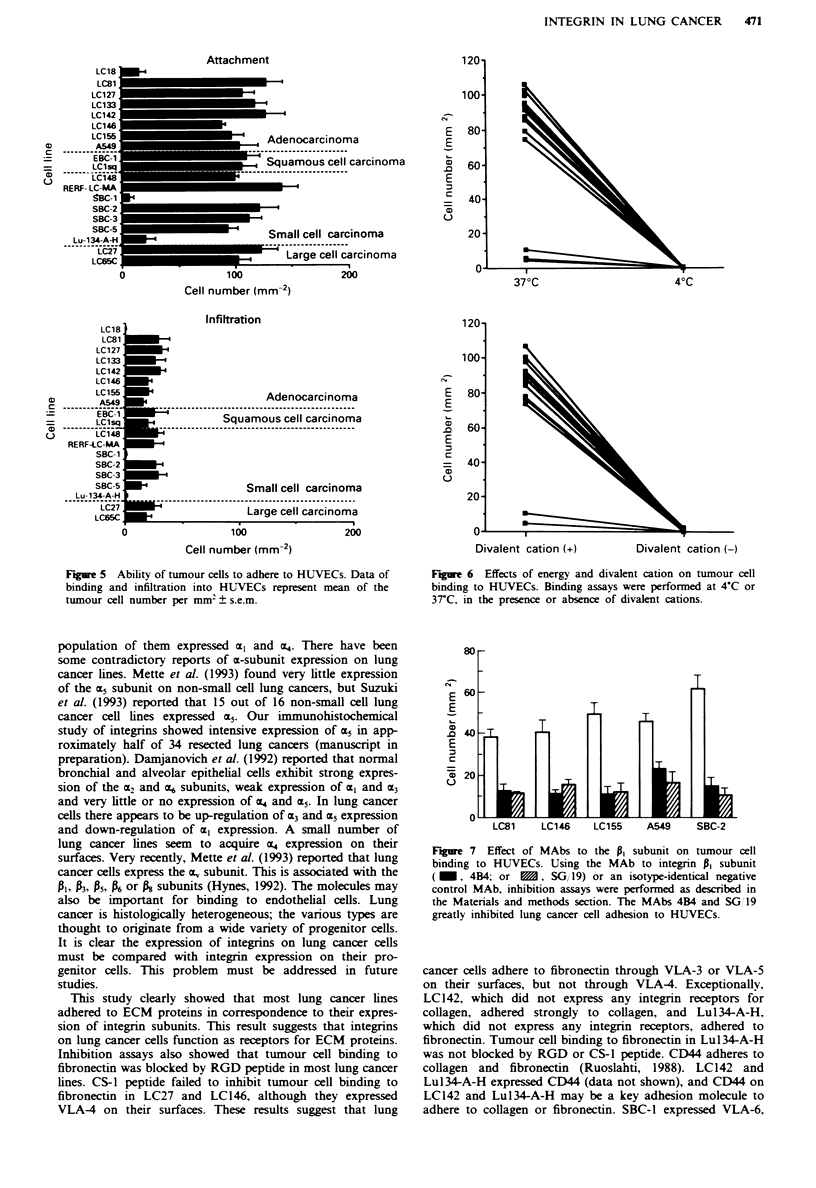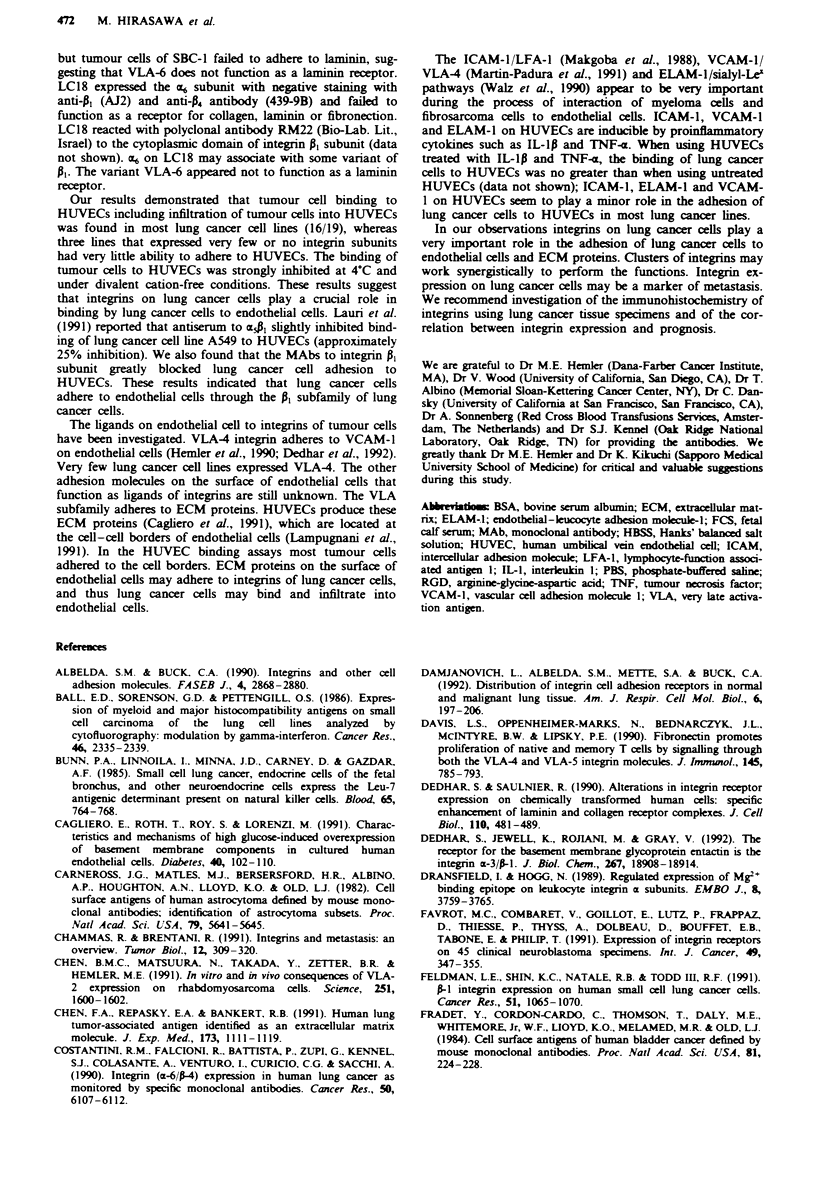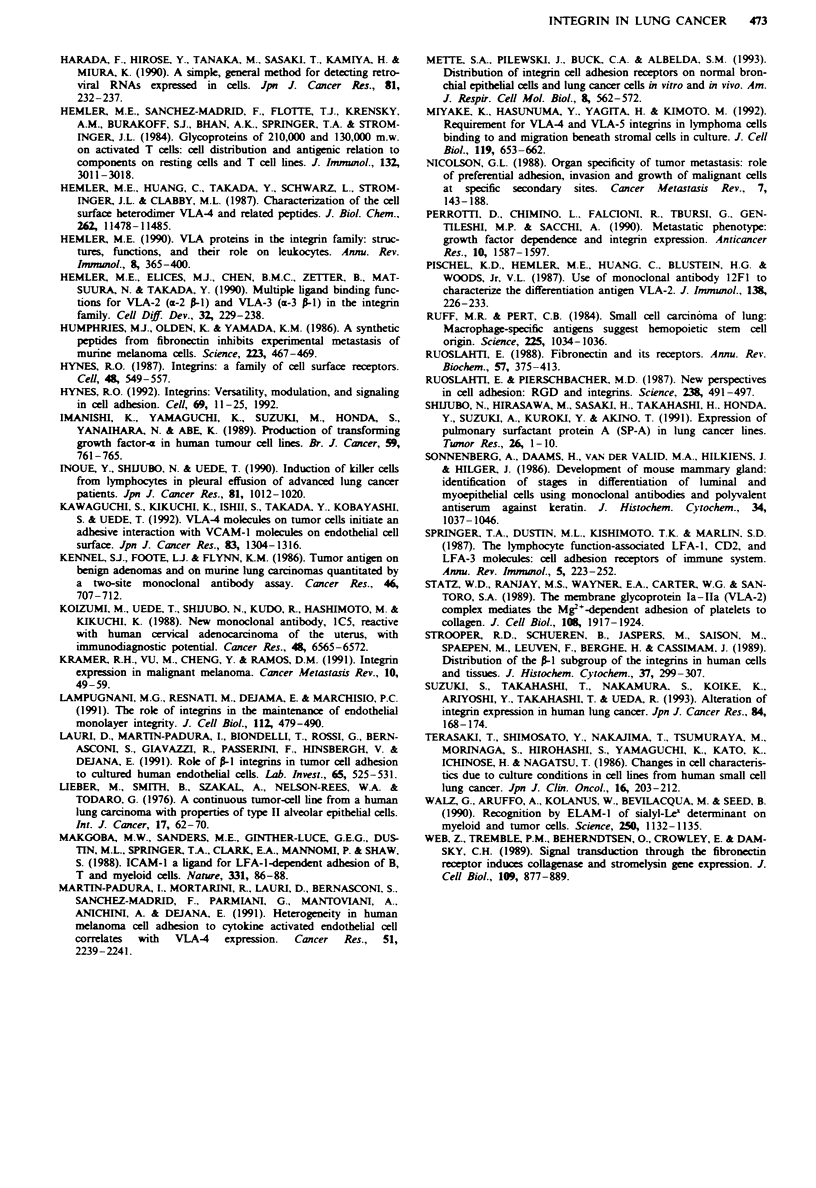# Integrin expression and ability to adhere to extracellular matrix proteins and endothelial cells in human lung cancer lines.

**DOI:** 10.1038/bjc.1994.329

**Published:** 1994-09

**Authors:** M. Hirasawa, N. Shijubo, T. Uede, S. Abe

**Affiliations:** Third Department of Internal Medicine, Sapporo Medical University School of Medicine, Japan.

## Abstract

**Images:**


Br. J. Cancer (1994), 70, 466–473

© Macmillan Press Ltd., 1994

### Integrin expression and ability to adhere to extracellular matrix proteins and endothelial cells in human lung cancer lines*

M. Hirasawa[1], N. Shijubo[1], T. Uede[2] & S. Abe[1]

[1]*Third Department of Internal Medicine, Sapporo Medical University School of Medicine;* [2]*Section of Immunopathogenesis, Institute of Immunological Science, Hokkaido University, Sapporo, Japan.*

**Summary** We examined the integrin expression in 19 human lung cancer cell lines with monoclonal antibodies to the integrin subunits $\alpha_1$, $\alpha_2$, $\alpha_3$, $\alpha_4$, $\alpha_5$, $\alpha_6$, $\beta_1$, $\beta_2$, and $\beta_4$. We measured their ability to adhere to the extracellular matrix (ECM) and human umbilical vein endothelial cells (HUVECs). Almost all lines expressed the $\beta_1$ subunit and approximately half of the lines expressed the $\beta_4$ subunit; by contrast, none expressed the $\beta_2$ subunit. Subunits $\alpha_2$, $\alpha_3$, $\alpha_5$ and $\alpha_6$ were frequently expressed, whereas very few lines expressed $\alpha_1$ and $\alpha_4$. Most lines adhered strongly to ECM (type I collagen, laminin and fibronectin) in correspondence to their expression of integrins. Binding by most lines to fibronectin was completely inhibited by arginine-glycine-aspartic acid (RGD) peptide. Three lines that expressed few or no integrins had very weak ability to adhere to ECM. Strong binding to HUVECs was found in most lines, but the three lines had very little ability to adhere to HUVECs. Binding to HUVECs was strongly inhibited at 4°C, under divalent cation-free conditions and by antibodies to the $\beta_1$ subunit. These results suggest that lung cancer cells adhere to ECM and endothelial cells through integrins, especially the $\beta_1$ subfamily.

Metastasis is the major cause of mortality in patients with malignant tumours. It consists of a series of events, of which the most important are the detachment of cancer cells, their migration to and then transportation by the circulation, adhesion to vascular endothelial cells, migration through the vascular wall and, finally, proliferation in the parenchyma of the target organs (Nicolson, 1988). The interaction of tumour cells with vascular endothelial cells is thought to be one of the most important steps in haematogenous metastasis (Albelda & Buck, 1990; Chammas & Brentani, 1991).

A dramatic step forward in the analysis of this interaction was taken with the identification of integrins, a family of adhesion molecules. The integrins are transmembrane glycoproteins that form heterodimers consisting of non-covalently associated α- and β-subunits (Hynes, 1987; Hemler, 1990). The majority of integrins can be grouped into several subfamilies defined by the presence of a common β-subunit. Many receptors for ECM proteins including the integrin family have been found on tumours (Ruoslahti & Pierschbacher, 1987). In the first step of their interaction with endothelial cells, melanoma (Martin-Padura *et al.*, 1991) and fibrosarcoma cells (Kawaguchi *et al.*, 1992) adhere to endothelial cells through VLA-4/VCAM-1 interaction. There have been several studies on integrin expression of lung cancers (Damjanovich *et al.*, 1992; Mette *et al.*, 1993; Suzuki *et al.*, 1993) and the ability of lung cancer cells to adhere to ECM proteins (Mette *et al.*, 1993). The key molecules by which lung cancer cells interact with endothelial cells remain unclear. We have examined the integrin expression of lung cancer cells and their ability to adhere to ECM proteins and endothelial cells using 19 lung cancer cell lines. The presence of integrins on lung cancer cells seems to be necessary for lung cancer cells to adhere to endothelial cells or ECM proteins. We discuss the role of integrins on lung cancer cells during the process of metastasis.

Correspondence: M. Hirasawa, Third Department of Internal Medicine, Sapporo Medical University School of Medicine, S-1, W-16, Chuo-ku, Sapporo 060, Japan.

*This investigation was supported in part by a Grant in Aid from the Ministry of Health and Welfare, Japan.

Received 14 December 1993; accepted in revised form 6 April 1994.

### Materials and methods

### *Lung cancer cell lines*

Nineteen lung cancer cell lines were used in this study. LC18, LC27, LC81, LC127, LC133, LC142, LC146, LC148 and LC155 were established in our laboratory (Inoue *et al.*, 1990; Shijubo *et al.*, 1991) and A549, EBC-1, LC1sq, RERF-LC-MA, SBC-1, SBC-2, SBC-3, SBC-5, Lu134-A-H and LC65C (Lieber *et el.*, 1976; Terasaki *et al.*, 1986; Imanishi *et al.*, 1989; Harada *et al.*, 1990) were supplied by the Japan Cancer Research Resources Bank. LC18, LC81, LC127, LC133, LC142, LC146, LC155 and A549 were established from lung adenocarcinoma, EBC-1 and LC1-sq from lung squamous cell carcinoma, LC148, Lu134-A-H, RERF-LC-MA, SBC-1, SBC-2, SBC-3 and SBC-5 from small-cell lung carcinoma and LC27 and LC65C from large cell lung carcinoma. These cell lines were adherent and maintained in RPMI-1640 medium supplemented with $2 \times 10^{-3}$ M L-glutamine, 10% fetal calf serum (FCS), 100 U ml$^{-1}$ penicillin and 100 μg ml$^{-1}$ streptomycin (hereafter referred to as the 'complete medium'). For binding assays, tumour cells were treated with PBS, pH 7.4, containing 0.05% trypsin (Sigma, St Louis, MO, USA) and 0.02% EDTA, collected, washed three times with PBS and resuspended in complete medium. The viability of tumour cells was more than 95% as judged from the ability to exclude trypan blue.

### *Reagents*

Human fibronection, laminin and collagen type I were purchased from Chemicon (Temecula, CA, USA). The ECM proteins were dissolved in $Ca^{2+}$, $Mg^{2+}$-free PBS. RGD-containing peptide (GRGDSP) and III CS CS-1 peptide (CDELPQLVTLPNLHGPEILDVPST) were purchased from Iwakiglass Institute, (Tokyo, Japan). RGD peptide is the recognition site of VLA-3 and VLA-5, and III CS CS-1 peptide is the recognition site of VLA-4 (Hynes, 1992).

### *Detection of integrin expression on lung cancer cells of lines*

Phenotyping of lung cancer cell lines was performed by indirect immunofluorescence using saturating amounts of MAbs to integrin subunits. These are listed in Table I. Normal mouse or rat IgG was used as a negative control.

Flow cytometric analysis was carried out using a FACScan (Becton Dickinson, San Jose, CA, USA). The fraction of positive cells after background subtraction was recorded for each sample study.

### *Binding assays to ECM proteins*

Each well of a 48-well culture plate (Costar, Cambridge, MA, USA) was pretreated with 300 μl of ECM protein solution (collagen type I, 100 μg $ml^{-1}$; laminin, 10 μg $ml^{-1}$; and fibronection, 10 μg $ml^{-1}$) at 4°C overnight. After washing three times with PBS containing 5 mg $ml^{-1}$ bovine serum albumin (BSA), the remaining uncoated sites were blocked by incubation with RPMI-1640 containing 5 mg $ml^{-1}$ BSA at 4°C for 4 h. Twelve thousand tumour cells of each line in 300 μl of RPMI-1640 containing 1% BSA were added to each well and then incubated at 37°C in 5% carbon dioxide for 2 h. After incubation, culture supernatant with unbound cells was removed by gentle aspiration and then washed three times with RPMI-1640 containing 1% BSA prewarmed to 37°C. Interactions between tumour cells and ECM proteins were examined under phase-contrast microscopy in six random fields at a magnification of × 40. Data represent the number of tumour cells binding to each ECM protein per $mm^2$ ± s.e.m.

Inhibition assays were performed to assess the effect of the synthetic fragments of fibronectin. Tumour cells pretreated with 100 μg $ml^{-1}$ RGD or 100 μg $ml^{-1}$ CS-1 peptide for 1 h were added directly to the wells. The binding assays were carried out as described above.

**Table I** Anti-integrin MAb used

| *Subunit* | *MAb name* | *Source* | *Reference* |
|---|---|---|---|
| $\alpha_1$ | TS2 7 | Dr Martin E. Hemler | Hemler *et al.* (1984) |
| $\alpha_2$ | 12F1 | Dr Virgil Wood | Pischel *et al.* (1987) |
| $\alpha_3$ | J134 | Dr Tony Albino | Fradit *et al.* (1984) |
| $\alpha_4$ | 8F2 | Dr Martin Hemler | Hemler *et al.* (1987) |
| $\alpha_5$ | BIIG2 | Dr Caroline Damsky | Web *et al.* (1989) |
| $\alpha_6$ | GoH3 | Dr Arnoud Sonnenberg | Sonnenberg *et al.* (1986) |
| $\beta_1$ | AJ2 | Dr Tony Albino | Carneross *et al.* (1982) |
| | 4B4 | Coulter | Davis *et al.* (1990) |
| | SG 19 | Seikagaku Corp. | Miyake *et al.* (1992) |
| $\beta_2$ | L130 | Becton Dickinson | Springer *et al.* (1987) |
| $\beta_2$ | 439-9B | Dr Stephen J. Kennel | Kennel *et al.* (1986) |

### *Binding assays to endothelial cells*

HUVECs were purchased from Kurabo (Osaka, Japan). HUVECs were cultured in eight-well Lab-Tek chambers (Nunc, Naperville, IL, USA) using a medium of endothelial cells, E-GM UV (Kurabo), at 37°C in 5% carbon dioxide to the subconfluent condition and then gently washed three times with complete medium. Twelve thousand tumour cells of each lung cancer line in 300 μl of complete medium were placed in the Lab-Tek chambers and incubated at 37°C in 5% carbon dioxide for 12 h. After incubation, chamber structures of Lab-Tek slides were removed and slide glasses with silicone gaskets were dipped face down into 50 ml of washing medium (RPMI-1640 containing 1% FCS) pre-warmed to 37°C, and washed under gentle agitation for 5 min. The chambers were then dried and fixed with methanol for 3 min. The chambers were stained with Diff-Quick (Kokusai Reagent, Kobe, Japan), which is a modification of the Wright and Giemsa staining method. The degree of binding and infiltration of tumour cells into HUVECs was evaluated under light microscopic examination in six random fields at a magnification of × 200. Halo-like images showing tumour cells infiltrated into HUVECs were assessed by phase-contrast illumination of the same field. Data represent means of tumour cell binding and infiltration per $mm^2$ ± s.e.m.

Assays were performed at 4°C or 37°C for 2 h to investigate the extent to which binding is dependent on energy. Next, we performed the assays under conditions of 1 mM

**Figure 1** Integrin expression on lung cancer cell lines. Integrin expression was determined by immunofluorescence using a panel of monoclonal antibodies against various integrin subunits (Table I). The fraction of positive cells after background subtraction was recorded for each sample study.

**Figure 2** Integrin expression of lung cancer cells with regard to receptors to ECM proteins [collagen (**a**) laminin (**b**) and fibronectin (**c**)] and ability of tumour cells to adhere to ECM proteins. Percentages of stained cells are divided into four distinct levels: -, very low (<5%); O, low (≥5% and <30%); ◉, moderate (≥ 30% and <60%); ●, high expression (≥ 60%). Data are expressed as mean number of tumour cells binding to ECM proteins per mm$^2$ ± s.e.m., as described in Materials and methods section.

$Ca^{2+}$, $Mg^{2+}$ HBSS or $Ca^{2+}$, $Mg^{2+}$-free HBSS to investigate divalent cation dependency of tumour cell binding to endothelial cells. These media were supplemented with 20% FCS dialysed three times against PBS for 12 h. The degree of binding to endothelial cells was evaluated by the same procedure as mentioned above.

Inhibition assays were performed to evaluate the effect of MAbs to integrin $\beta_1$ subunit on tumour cell binding to endothelial cells. Five lung cancer cell lines (LC81, LC146, LC155, A549 and SBC-2) were preincubated with MAbs [4B4 or SG/19 (40 $\mu g\ ml^{-1}$)] to integrin $\beta_1$ subunit or with an isotype-identical negative control MAb (1C5) to human cervical adenocarcinoma (Koizumi *et al.*, 1988) for 1 h. Following preincubation with the MAbs, 12,000 tumour cells were added directly to the chambers without washing. The binding assays were carried out as described above. Incubation time of the assays was 1 h.

Another series of inhibition assays was performed to assess the effect of the synthetic fragments of fibronectin. The tumour cells were preincubated with 100 $\mu g\ ml^{-1}$ RGD or 100 $\mu g\ ml^{-1}$ CS-1 peptide at 37°C in 5% carbon dioxide for 1 h. Following preincubation with the synthetic fragments, the tumour cells were added directly to the chambers without washing. Assays were carried out as described above, except that the incubation time was 30 min.

### Results

### *Expression of integrins in lung cancer lines*

We examined integrin expression on tumour cells of 19 lung cancer cell lines using MAbs (Figure 1). Integrin expression on tumour cells of lung cancer lines is quite heterogeneous. There was no typical feature of the expression of integrin subunits on any histological cell types of lung cancer cell lines. The degree of expression of the $\beta$-subunit varied, however almost all cell lines (17 out of 19) expressed the $\beta_1$ subunit, and 9 out of 19 lines expressed the $\beta_4$ subunit. By contrast none of them expressed $\beta_2$. The $\alpha$-subunits most consistently found on tumour cells of lung cancer lines were $\alpha_2$ (10/19), $\alpha_3$ (14/19), $\alpha_5$ (15/19) and $\alpha_6$ (15/19). A very minor population of lung cancer lines had $\alpha_1$ (1/19) and $\alpha_4$ subunits (2/19).

### *Ability of lung cancer cells to adhere to ECM proteins*

Since the integrins are known to function as adhesion receptors for ECM proteins, we measured the adhesive properties of the tumour cells in a cell attachment assay. The expressions of the integrin subunits which function as receptors to ECM proteins and their properties of adhesion to ECM proteins are shown in Figure 2. Fourteen of 19 lung cancer lines had at least one of the integrin subunits that are collagen receptors (VLA-1, VLA-2 and VLA-3) and were able to adhere to collagen strongly. No VLA-1, VLA-2 or VLA-3 was found in five lines (LC18, LC142, SBC-1, Lu134-A-H and LC27). LC142 adhered strongly to collagen, but cells of the remaining four lines failed to adhere to collagen. Seventeen of 19 lung cancer lines expressed at least one of the integrin subunits as laminin receptors (VLA-1, VLA-2, VLA-3, VLA-6 and $\alpha_6\ \beta_4$). Nearly all of the lines (16/17) with integrin laminin receptors were able to adhere strongly to laminin. SBC-1 cells expressed VLA-6, but failed to adhere to laminin. LC18 expressed only the $\alpha_6$ subunit but neither the $\beta_1$ nor $\beta_4$ subunit, and Lu134-A-H expressed none of the integrin subunits examined in this study. LC18 and Lu134-A-H failed to adhere to laminin. Sixteen of 19 lung cancer lines expressed at least one of the integrin subunits that function as fibronectin receptors (VLA-3, VLA-4 and VLA-5). Lung cancer cells of these lines adhered strongly to fibronectin. LC18, SBC-1 and Lu134-A-H never expressed VLA-3, VLA-4 or VLA-5. LC18 and SBC-1 failed to adhere to fibronectin, whereas Lu134-A-H bound strongly to fibronectin.

### *Binding inhibition of lung cancer cells to fibronectin using RGD or CS-1 peptide*

Binding inhibition assays to fibronectin with RGD or CS-1 peptide were performed using the 17 lung cancer lines that had adhered strongly to fibronectin (Figure 3). RGD peptide inhibited the binding of tumour cells to fibronection (Figure 3). RGD peptide inhibited the binding of tumour cells to fibronectin in nearly all of these lung cancer lines (16/17), but showed no such action with Lu134-A-H. CS-1 peptide failed to block binding of tumour cells to fibronectin in all examined lines.

### *Ability of tumour cells to adhere to HUVECs*

We examined the ability of lung cancer cells to adhere to and infiltrate into HUVECs. A typical interaction is shown in Figure 4. Figure 4a depicts a case of low binding (by SBC-1) to the HUVECs' surface, and Figure 4b shows strong binding (by LC27). Figure 4c depicts a halo-like image showing the tumour cell (LC27) infiltrated into the HUVEC, and Figure 4d shows simply adhesion of tumour cells (LC155) to the HUVECs' surface. Data on binding and infiltration by tumour cells are shown in Figure 5. Tumour cells of most lung cancer lines showed strong binding to HUVECs and frequently infiltrated into them. The three lines LC18, SBC-1 and Lu134-A-H had very weak binding to HUVECs and seldom infiltration.

### *Adhesion assays at 4°C, and under divalent cation-free conditions*

Since integrins are known to require energy (Dransfield & Hogg, 1989) and divalent cations (Statz *et al.*, 1989), we

**Figure 3** Effect of synthetic fragments (RGD and CS-1 peptide) of fibronectin on the adhesion of tumour cells to fibronectin. Inhibition assays were performed as described in the Materials and methods section and data are expressed as mean number of tumour cells binding to fibronectin per $mm^2 \pm$ s.e.m.

Figure 4 Representative microphotographs of interactive behaviour between tumour cells and HUVECs. a shows weak (SBC-1) and b shows strong binding of tumour cells (LC27). c depicts a halo-like image (arrow) showing the tumour cell (LC27) infiltrated into the HUVEC and d depicts simple adhesion of tumour cells (LC155). Original magnification: a and b, × 235; and c and d × 470.

performed adhesion assays on HUVECs at 4°C or at 37°C and under conditions with or without divalent cations (Figure 6). Tumour cell binding to HUVECs was much lower at 4°C or under divalent cation-free conditions than at 37°C or in the presence of divalent cations.

*Inhibition of lung cancer cell adhesion to HUVECs by RGD, CS-1 peptide or MAbs to integrin $\beta_1$ subunit.*

Inhibition assays to HUVECs with the MAbs (4B4 or SG/19) to the $\beta_1$ subunit were performed (Figure 7). Both MAbs showed great inhibition of adhesion ranging from 51% to 83% inhibition, in comparison with the binding with an isotype-matched negative control MAb. Next, assays were performed using RGD or CS-1 peptide; assays to these failed to inhibit binding of tumour cells to HUVECs in all examined lines (data not shown).

### Discussion

Integrins mediate ECM–cell or cell–cell interactions (Hemler, 1990*a*). Embryonic development, maintenance of tissue architecture, inflammatory response and wound healing all involve the interaction of cells with ECM proteins or neighbouring cells; thus, the integrins play a very important role in mediating such events (Hynes, 1987) and the metastasis of malignant tumours (Chammas & Brentani, 1991). Metastatic subtypes of Lewis lung carcinoma expressed $\alpha_6\ \beta_4$ more frequently than did non-metastatic subtypes (Perrotti *et al*., 1990). Rhabdomyosarcoma cells transfected with genes from VLA-2 became substantially more metastatic than the original tumour cells (B.M.C. Chen *et al*., 1991). Both antibodies to integrins and synthetic peptides of ECM proteins prevented the formation of the lung nodules in mice injected with B16 melanoma cells (Humphries *et al*., 1986). There is increasing evidence that neoplastic transformation is associated with modification of integrin expression (Dedhar & Saulnier, 1990). Human lung cancers expressed at least 20 times more $\alpha_2$ subunit RNA message than normal adult human lung tissue (F.A. Chen *et al*., 1991). Modification of integrin expression may affect the ability to metastasise.

Integrin expression in tumours such as melanoma (Kramer *et al*., 1991), neuroblastoma (Favrot *et al*., 1991) and osteosarcoma (Lauri *et al*., 1991) has been extensively studied. Several studies have also focused on lung cancers (Damjanovich *et al*., 1992; Mette *et al*., 1993; Suzuki *et al*., 1993), but have not clearly shown distinct patterns of integrin expression among various histological types of lung cancer.

Most lung cancer cell lines as well as normal bronchial epithelial cells (Strooper *et al*., 1989) appear to express the $\beta_1$ subunit (Feldman *et al*., 1991). Although several investigators (Ruff & Pert, 1984; Bunn *et al*., 1985; Ball *et al*., 1986) have reported the $\alpha_M$ subunit in a small number of small cell lung cancer lines, very little expression of the $\beta_2$ subunit was found on small cell lung cancer cells (Feldman *et al*., 1991). Our data from six small cell lung cancer lines were in agreement with the result of Feldman *et al*., but all lung cancer cell lines we examined, regardless of histological type, showed zero expression of the $\beta_2$ subunit on their surfaces.

Costantini *et al*. (1990) reported that expression of the $\beta_4$ subunit occurs in non-small cell lung cancers, but not in small cell lung cancers. In our study, $\beta_4$ was expressed in 7 of 12 non small cell lung cancer lines and in two of seven small-cell lung cancer lines.

This study showed that the α-subunits most consistently found on lung cancer cells were $\alpha_2$, $\alpha_3$, $\alpha_5$ and $\alpha_6$; a minor

**Figure 5** Ability of tumour cells to adhere to HUVECs. Data of binding and infiltration into HUVECs represent mean of the tumour cell number per mm$^2$ $\pm$ s.e.m.

**Figure 6** Effects of energy and divalent cation on tumour cell binding to HUVECs. Binding assays were performed at 4°C or 37°C, in the presence or absence of divalent cations.

**Figure 7** Effect of MAbs to the $\beta_1$ subunit on tumour cell binding to HUVECs. Using the MAb to integrin $\beta_1$ subunit (■, 4B4; or ▨, SG/19) or an isotype-identical negative control MAb, inhibition assays were performed as described in the Materials and methods section. The MAbs 4B4 and SG/19 greatly inhibited lung cancer cell adhesion to HUVECs.

population of them expressed $\alpha_1$ and $\alpha_4$. There have been some contradictory reports of $\alpha$-subunit expression on lung cancer lines. Mette *et al.* (1993) found very little expression of the $\alpha_5$ subunit on non-small cell lung cancers, but Suzuki *et al.* (1993) reported that 15 out of 16 non-small cell lung cancer cell lines expressed $\alpha_5$. Our immunohistochemical study of integrins showed intensive expression of $\alpha_5$ in approximately half of 34 resected lung cancers (manuscript in preparation). Damjanovich *et al.* (1992) reported that normal bronchial and alveolar epithelial cells exhibit strong expression of the $\alpha_2$ and $\alpha_6$ subunits, weak expression of $\alpha_1$ and $\alpha_3$ and very little or no expression of $\alpha_4$ and $\alpha_5$. In lung cancer cells there appears to be up-regulation of $\alpha_3$ and $\alpha_5$ expression and down-regulation of $\alpha_1$ expression. A small number of lung cancer lines seem to acquire $\alpha_4$ expression on their surfaces. Very recently, Mette *et al.* (1993) reported that lung cancer cells express the $\alpha_v$ subunit. This is associated with the $\beta_1$, $\beta_3$, $\beta_5$, $\beta_6$ or $\beta_8$ subunits (Hynes, 1992). The molecules may also be important for binding to endothelial cells. Lung cancer is histologically heterogeneous; the various types are thought to originate from a wide variety of progenitor cells. It is clear the expression of integrins on lung cancer cells must be compared with integrin expression on their progenitor cells. This problem must be addressed in future studies.

This study clearly showed that most lung cancer lines adhered to ECM proteins in correspondence to their expression of integrin subunits. This result suggests that integrins on lung cancer cells function as receptors for ECM proteins. Inhibition assays also showed that tumour cell binding to fibronectin was blocked by RGD peptide in most lung cancer lines. CS-1 peptide failed to inhibit tumour cell binding to fibronectin in LC27 and LC146, although they expressed VLA-4 on their surfaces. These results suggest that lung cancer cells adhere to fibronectin through VLA-3 or VLA-5 on their surfaces, but not through VLA-4. Exceptionally, LC142, which did not express any integrin receptors for collagen, adhered strongly to collagen, and Lu134-A-H, which did not express any integrin receptors, adhered to fibronectin. Tumour cell binding to fibronectin in Lu134-A-H was not blocked by RGD or CS-1 peptide. CD44 adheres to collagen and fibronectin (Ruoslahti, 1988). LC142 and Lu134-A-H expressed CD44 (data not shown), and CD44 on LC142 and Lu134-A-H may be a key adhesion molecule to adhere to collagen or fibronectin. SBC-1 expressed VLA-6, but tumour cells of SBC-1 failed to adhere to laminin, suggesting that VLA-6 does not function as a laminin receptor. LC18 expressed the $\alpha_6$ subunit with negative staining with anti-$\beta_1$ (AJ2) and anti-$\beta_4$ antibody (439-9B) and failed to function as a receptor for collagen, laminin or fibronection. LC18 reacted with polyclonal antibody RM22 (Bio-Lab. Lit., Israel) to the cytoplasmic domain of integrin $\beta_1$ subunit (data not shown). $\alpha_6$ on LC18 may associate with some variant of $\beta_1$. The variant VLA-6 appeared not to function as a laminin receptor.

Our results demonstrated that tumour cell binding to HUVECs including infiltration of tumour cells into HUVECs was found in most lung cancer cell lines (16/19), whereas three lines that expressed very few or no integrin subunits had very little ability to adhere to HUVECs. The binding of tumour cells to HUVECs was strongly inhibited at 4°C and under divalent cation-free conditions. These results suggest that integrins on lung cancer cells play a crucial role in binding by lung cancer cells to endothelial cells. Lauri et al. (1991) reported that antiserum to $\alpha_5\beta_1$ slightly inhibited binding of lung cancer cell line A549 to HUVECs (approximately 25% inhibition). We also found that the MAbs to integrin $\beta_1$ subunit greatly blocked lung cancer cell adhesion to HUVECs. These results indicated that lung cancer cells adhere to endothelial cells through the $\beta_1$ subfamily of lung cancer cells.

The ligands on endothelial cell to integrins of tumour cells have been investigated. VLA-4 integrin adheres to VCAM-1 on endothelial cells (Hemler et al., 1990; Dedhar et al., 1992). Very few lung cancer cell lines expressed VLA-4. The other adhesion molecules on the surface of endothelial cells that function as ligands of integrins are still unknown. The VLA subfamily adheres to ECM proteins. HUVECs produce these ECM proteins (Cagliero et al., 1991), which are located at the cell-cell borders of endothelial cells (Lampugnani et al., 1991). In the HUVEC binding assays most tumour cells adhered to the cell borders. ECM proteins on the surface of endothelial cells may adhere to integrins of lung cancer cells, and thus lung cancer cells may bind and infiltrate into endothelial cells.

The ICAM-1/LFA-1 (Makgoba et al., 1988), VCAM-1/VLA-4 (Martin-Padura et al., 1991) and ELAM-1/sialyl-Le$^x$ pathways (Walz et al., 1990) appear to be very important during the process of interaction of myeloma cells and fibrosarcoma cells to endothelial cells. ICAM-1, VCAM-1 and ELAM-1 on HUVECs are inducible by proinflammatory cytokines such as IL-1$\beta$ and TNF-$\alpha$. When using HUVECs treated with IL-1$\beta$ and TNF-$\alpha$, the binding of lung cancer cells to HUVECs was no greater than when using untreated HUVECs (data not shown); ICAM-1, ELAM-1 and VCAM-1 on HUVECs seem to play a minor role in the adhesion of lung cancer cells to HUVECs in most lung cancer lines.

In our observations integrins on lung cancer cells play a very important role in the adhesion of lung cancer cells to endothelial cells and ECM proteins. Clusters of integrins may work synergistically to perform the functions. Integrin expression on lung cancer cells may be a marker of metastasis. We recommend investigation of the immunohistochemistry of integrins using lung cancer tissue specimens and of the correlation between integrin expression and prognosis.

We are grateful to Dr M.E. Hemler (Dana-Farber Cancer Institute, MA), Dr V. Wood (University of California, San Diego, CA), Dr T. Albino (Memorial Sloan-Kettering Cancer Center, NY), Dr C. Dansky (University of California at San Francisco, San Francisco, CA), Dr A. Sonnenberg (Red Cross Blood Transfusions Services, Amsterdam, The Netherlands) and Dr S.J. Kennel (Oak Ridge National Laboratory, Oak Ridge, TN) for providing the antibodies. We greatly thank Dr M.E. Hemler and Dr K. Kikuchi (Sapporo Medical University School of Medicine) for critical and valuable suggestions during this study.

**Abbreviations:** BSA, bovine serum albumin; ECM, extracellular matrix; ELAM-1; endothelial-leucocyte adhesion molecule-1; FCS, fetal calf serum; MAb, monoclonal antibody; HBSS, Hanks' balanced salt solution; HUVEC, human umbilical vein endothelial cell; ICAM, intercellular adhesion molecule; LFA-1, lymphocyte-function associated antigen 1; IL-1, interleukin 1; PBS, phosphate-buffered saline; RGD, arginine-glycine-aspartic acid; TNF, tumour necrosis factor; VCAM-1, vascular cell adhesion molecule 1; VLA, very late activation antigen.

### References

ALBELDA, S.M. & BUCK, C.A. (1990). Integrins and other cell adhesion molecules. *FASEB J.*, **4**, 2868-2880.

BALL, E.D., SORENSON, G.D. & PETTENGILL, O.S. (1986). Expression of myeloid and major histocompatibility antigens on small cell carcinoma of the lung cell lines analyzed by cytofluorography: modulation by gamma-interferon. *Cancer Res.*, **46**, 2335-2339.

BUNN, P.A., LINNOILA, I., MINNA, J.D., CARNEY, D. & GAZDAR, A.F. (1985). Small cell lung cancer, endocrine cells of the fetal bronchus, and other neuroendocrine cells express the Leu-7 antigenic determinant present on natural killer cells. *Blood*, **65**, 764-768.

CAGLIERO, E., ROTH, T., ROY, S. & LORENZI, M. (1991). Characteristics and mechanisms of high glucose-induced overexpression of basement membrane components in cultured human endothelial cells. *Diabetes*, **40**, 102-110.

CARNEROSS, J.G., MATLES, M.J., BERSERSFORD, H.R., ALBINO, A.P., HOUGHTON, A.N., LLOYD, K.O. & OLD, L.J. (1982). Cell surface antigens of human astrocytoma defined by mouse monoclonal antibodies; identification of astrocytoma subsets. *Proc. Natl Acad. Sci. USA*, **79**, 5641-5645.

CHAMMAS, R. & BRENTANI, R. (1991). Integrins and metastasis: an overview. *Tumor Biol.*, **12**, 309-320.

CHEN, B.M.C., MATSUURA, N., TAKADA, Y., ZETTER, B.R. & HEMLER, M.E. (1991). *In vitro* and *in vivo* consequences of VLA-2 expression on rhabdomyosarcoma cells. *Science*, **251**, 1600-1602.

CHEN, F.A., REPASKY, E.A. & BANKERT, R.B. (1991). Human lung tumor-associated antigen identified as an extracellular matrix molecule. *J. Exp. Med.*, **173**, 1111-1119.

COSTANTINI, R.M., FALCIONI, R., BATTISTA, P., ZUPI, G., KENNEL, S.J., COLASANTE, A., VENTURO, I., CURICIO, C.G. & SACCHI, A. (1990). Integrin (α-6/β-4) expression in human lung cancer as monitored by specific monoclonal antibodies. *Cancer Res.*, **50**, 6107-6112.

DAMJANOVICH, L., ALBELDA, S.M., METTE, S.A. & BUCK, C.A. (1992). Distribution of integrin cell adhesion receptors in normal and malignant lung tissue. *Am. J. Respir. Cell Mol. Biol.*, **6**, 197-206.

DAVIS, L.S., OPPENHEIMER-MARKS, N., BEDNARCZYK, J.L., MCINTYRE, B.W. & LIPSKY, P.E. (1990). Fibronectin promotes proliferation of native and memory T cells by signalling through both the VLA-4 and VLA-5 integrin molecules. *J. Immunol.*, **145**, 785-793.

DEDHAR, S. & SAULNIER, R. (1990). Alterations in integrin receptor expression on chemically transformed human cells: specific enhancement of laminin and collagen receptor complexes. *J. Cell Biol.*, **110**, 481-489.

DEDHAR, S., JEWELL, K., ROJIANI, M. & GRAY, V. (1992). The receptor for the basement membrane glycoprotein entactin is the integrin α-3/β-1. *J. Biol. Chem.*, **267**, 18908-18914.

DRANSFIELD, I. & HOGG, N. (1989). Regulated expression of $Mg^{2+}$ binding epitope on leukocyte integrin α subunits. *EMBO J.*, **8**, 3759-3765.

FAVROT, M.C., COMBARET, V., GOILLOT, E., LUTZ, P., FRAPPAZ, D., THIESSE, P., THYSS, A., DOLBEAU, D., BOUFFET, E.B., TABONE, E. & PHILIP, T. (1991). Expression of integrin receptors on 45 clinical neuroblastoma specimens. *Int. J. Cancer*, **49**, 347-355.

FELDMAN, L.E., SHIN, K.C., NATALE, R.B. & TODD III, R.F. (1991). β-1 integrin expression on human small cell lung cancer cells. *Cancer Res.*, **51**, 1065-1070.

FRADET, Y., CORDON-CARDO, C., THOMSON, T., DALY, M.E., WHITEMORE, Jr, W.F., LIOYD, K.O., MELAMED, M.R. & OLD, L.J. (1984). Cell surface antigens of human bladder cancer defined by mouse monoclonal antibodies. *Proc. Natl Acad. Sci. USA*, **81**, 224-228.

HARADA, F., HIROSE, Y., TANAKA, M., SASAKI, T., KAMIYA, H. & MIURA, K. (1990). A simple, general method for detecting retroviral RNAs expressed in cells. *Jpn J. Cancer Res.*, **81**, 232-237.

HEMLER, M.E., SANCHEZ-MADRID, F., FLOTTE, T.J., KRENSKY, A.M., BURAKOFF, S.J., BHAN, A.K., SPRINGER, T.A. & STROMINGER, J.L. (1984). Glycoproteins of 210,000 and 130,000 m.w. on activated T cells: cell distribution and antigenic relation to components on resting cells and T cell lines. *J. Immunol.*, **132**, 3011-3018.

HEMLER, M.E., HUANG, C., TAKADA, Y., SCHWARZ, L., STROMINGER, J.L. & CLABBY, M.L. (1987). Characterization of the cell surface heterodimer VLA-4 and related peptides. *J. Biol. Chem.*, **262**, 11478-11485.

HEMLER, M.E. (1990). VLA proteins in the integrin family: structures, functions, and their role on leukocytes. *Annu. Rev. Immunol.*, **8**, 365-400.

HEMLER, M.E., ELICES, M.J., CHEN, B.M.C., ZETTER, B., MATSUURA, N. & TAKADA, Y. (1990). Multiple ligand binding functions for VLA-2 (α-2 β-1) and VLA-3 (α-3 β-1) in the integrin family. *Cell Diff. Dev.*, **32**, 229-238.

HUMPHRIES, M.J., OLDEN, K. & YAMADA, K.M. (1986). A synthetic peptides from fibronectin inhibits experimental metastasis of murine melanoma cells. *Science*, **223**, 467-469.

HYNES, R.O. (1987). Integrins: a family of cell surface receptors. *Cell*, **48**, 549-557.

HYNES, R.O. (1992). Integrins: Versatility, modulation, and signaling in cell adhesion. *Cell*, **69**, 11-25, 1992.

IMANISHI, K., YAMAGUCHI, K., SUZUKI, M., HONDA, S., YANAIHARA, N. & ABE, K. (1989). Production of transforming growth factor-α in human tumour cell lines. *Br. J. Cancer*, **59**, 761-765.

INOUE, Y., SHIJUBO, N. & UEDE, T. (1990). Induction of killer cells from lymphocytes in pleural effusion of advanced lung cancer patients. *Jpn J. Cancer Res.*, **81**, 1012-1020.

KAWAGUCHI, S., KIKUCHI, K., ISHII, S., TAKADA, Y., KOBAYASHI, S. & UEDE, T. (1992). VLA-4 molecules on tumor cells initiate an adhesive interaction with VCAM-1 molecules on endothelial cell surface. *Jpn J. Cancer Res.*, **83**, 1304-1316.

KENNEL, S.J., FOOTE, L.J. & FLYNN, K.M. (1986). Tumor antigen on benign adenomas and on murine lung carcinomas quantitated by a two-site monoclonal antibody assay. *Cancer Res.*, **46**, 707-712.

KOIZUMI, M., UEDE, T., SHIJUBO, N., KUDO, R., HASHIMOTO, M. & KIKUCHI, K. (1988). New monoclonal antibody, 1C5, reactive with human cervical adenocarcinoma of the uterus, with immunodiagnostic potential. *Cancer Res.*, **48**, 6565-6572.

KRAMER, R.H., VU, M., CHENG, Y. & RAMOS, D.M. (1991). Integrin expression in malignant melanoma. *Cancer Metastasis Rev.*, **10**, 49-59.

LAMPUGNANI, M.G., RESNATI, M., DEJAMA, E. & MARCHISIO, P.C. (1991). The role of integrins in the maintenance of endothelial monolayer integrity. *J. Cell Biol.*, **112**, 479-490.

LAURI, D., MARTIN-PADURA, I., BIONDELLI, T., ROSSI, G., BERNASCONI, S., GIAVAZZI, R., PASSERINI, F., HINSBERGH, V. & DEJANA, E. (1991). Role of β-1 integrins in tumor cell adhesion to cultured human endothelial cells. *Lab. Invest.*, **65**, 525-531.

LIEBER, M., SMITH, B., SZAKAL, A., NELSON-REES, W.A. & TODARO, G. (1976). A continuous tumor-cell line from a human lung carcinoma with properties of type II alveolar epithelial cells. *Int. J. Cancer*, **17**, 62-70.

MAKGOBA, M.W., SANDERS, M.E., GINTHER-LUCE, G.E.G., DUSTIN, M.L., SPRINGER, T.A., CLARK, E.A., MANNOMI, P. & SHAW, S. (1988). ICAM-1 a ligand for LFA-1-dependent adhesion of B, T and myeloid cells. *Nature*, **331**, 86-88.

MARTIN-PADURA, I., MORTARINI, R., LAURI, D., BERNASCONI, S., SANCHEZ-MADRID, F., PARMIANI, G., MANTOVIANI, A., ANICHINI, A. & DEJANA, E. (1991). Heterogeneity in human melanoma cell adhesion to cytokine activated endothelial cell correlates with VLA-4 expression. *Cancer Res.*, **51**, 2239-2241.

METTE, S.A., PILEWSKI, J., BUCK, C.A. & ALBELDA, S.M. (1993). Distribution of integrin cell adhesion receptors on normal bronchial epithelial cells and lung cancer cells *in vitro* and *in vivo*. *Am. J. Respir. Cell Mol. Biol.*, **8**, 562-572.

MIYAKE, K., HASUNUMA, Y., YAGITA, H. & KIMOTO, M. (1992). Requirement for VLA-4 and VLA-5 integrins in lymphoma cells binding to and migration beneath stromal cells in culture. *J. Cell Biol.*, **119**, 653-662.

NICOLSON, G.L. (1988). Organ specificity of tumor metastasis: role of preferential adhesion, invasion and growth of malignant cells at specific secondary sites. *Cancer Metastasis Rev.*, **7**, 143-188.

PERROTTI, D., CHIMINO, L., FALCIONI, R., TBURSI, G., GENTILESHI, M.P. & SACCHI, A. (1990). Metastatic phenotype: growth factor dependence and integrin expression. *Anticancer Res.*, **10**, 1587-1597.

PISCHEL, K.D., HEMLER, M.E., HUANG, C., BLUSTEIN, H.G. & WOODS, Jr, V.L. (1987). Use of monoclonal antibody 12F1 to characterize the differentiation antigen VLA-2. *J. Immunol.*, **138**, 226-233.

RUFF, M.R. & PERT, C.B. (1984). Small cell carcinoma of lung: Macrophage-specific antigens suggest hemopoietic stem cell origin. *Science*, **225**, 1034-1036.

RUOSLAHTI, E. (1988). Fibronectin and its receptors. *Annu. Rev. Biochem.*, **57**, 375-413.

RUOSLAHTI, E. & PIERSCHBACHER, M.D. (1987). New perspectives in cell adhesion: RGD and integrins. *Science*, **238**, 491-497.

SHIJUBO, N., HIRASAWA, M., SASAKI, H., TAKAHASHI, H., HONDA, Y., SUZUKI, A., KUROKI, Y. & AKINO, T. (1991). Expression of pulmonary surfactant protein A (SP-A) in lung cancer lines. *Tumor Res.*, **26**, 1-10.

SONNENBERG, A., DAAMS, H., VAN DER VALID, M.A., HILKIENS, J. & HILGER, J. (1986). Development of mouse mammary gland: identification of stages in differentiation of luminal and myoepithelial cells using monoclonal antibodies and polyvalent antiserum against keratin. *J. Histochem. Cytochem.*, **34**, 1037-1046.

SPRINGER, T.A., DUSTIN, M.L., KISHIMOTO, T.K. & MARLIN, S.D. (1987). The lymphocyte function-associated LFA-1, CD2, and LFA-3 molecules: cell adhesion receptors of immune system. *Annu. Rev. Immunol.*, **5**, 223-252.

STATZ, W.D., RANJAY, M.S., WAYNER, E.A., CARTER, W.G. & SANTORO, S.A. (1989). The membrane glycoprotein Ia-IIa (VLA-2) complex mediates the $Mg^{2+}$-dependent adhesion of platelets to collagen. *J. Cell Biol.*, **108**, 1917-1924.

STROOPER, R.D., SCHUEREN, B., JASPERS, M., SAISON, M., SPAEPEN, M., LEUVEN, F., BERGHE, H. & CASSIMAM, J. (1989). Distribution of the β-1 subgroup of the integrins in human cells and tissues. *J. Histochem. Cytochem.*, **37**, 299-307.

SUZUKI, S., TAKAHASHI, T., NAKAMURA, S., KOIKE, K., ARIYOSHI, Y., TAKAHASHI, T. & UEDA, R. (1993). Alteration of integrin expression in human lung cancer. *Jpn J. Cancer Res.*, **84**, 168-174.

TERASAKI, T., SHIMOSATO, Y., NAKAJIMA, T., TSUMURAYA, M., MORINAGA, S., HIROHASHI, S., YAMAGUCHI, K., KATO, K., ICHINOSE, H. & NAGATSU, T. (1986). Changes in cell characteristics due to culture conditions in cell lines from human small cell lung cancer. *Jpn J. Clin. Oncol.*, **16**, 203-212.

WALZ, G., ARUFFO, A., KOLANUS, W., BEVILACQUA, M. & SEED, B. (1990). Recognition by ELAM-1 of sialyl-$Le^x$ determinant on myeloid and tumor cells. *Science*, **250**, 1132-1135.

WEB, Z., TREMBLE, P.M., BEHERNDTSEN, O., CROWLEY, E. & DAMSKY, C.H. (1989). Signal transduction through the fibronectin receptor induces collagenase and stromelysin gene expression. *J. Cell Biol.*, **109**, 877-889.